# Arylsulfatase B Improves Locomotor Function after Mouse Spinal Cord Injury

**DOI:** 10.1371/journal.pone.0057415

**Published:** 2013-03-08

**Authors:** Myungsik Yoo, Muntasir Khaled, Kurt M. Gibbs, Jonghun Kim, Björn Kowalewski, Thomas Dierks, Melitta Schachner

**Affiliations:** 1 W. M. Keck Center for Collaborative Neuroscience and Department of Cell Biology and Neuroscience, Rutgers University, Piscataway, New Jersey, United States of America; 2 Department of Chemistry, Biochemistry I, Bielefeld University, Universitätsstr Bielefeld, Germany; 3 Center for Neuroscience, Shantou University Medical College, Shantou, Guandong Province, People’s Republic of China; University of Edinburgh, United Kingdom

## Abstract

Bacterial chondroitinase ABC (ChaseABC) has been used to remove the inhibitory chondroitin sulfate chains from chondroitin sulfate proteoglycans to improve regeneration after rodent spinal cord injury. We hypothesized that the mammalian enzyme arylsulfatase B (ARSB) would also enhance recovery after mouse spinal cord injury. Application of the mammalian enzyme would be an attractive alternative to ChaseABC because of its more robust chemical stability and reduced immunogenicity. A one-time injection of human ARSB into injured mouse spinal cord eliminated immunoreactivity for chondroitin sulfates within five days, and up to 9 weeks after injury. After a moderate spinal cord injury, we observed improvements of locomotor recovery assessed by the Basso Mouse Scale (BMS) in ARSB treated mice, compared to the buffer-treated control group, at 6 weeks after injection. After a severe spinal cord injury, mice injected with equivalent units of ARSB or ChaseABC improved similarly and both groups achieved significantly more locomotor recovery than the buffer-treated control mice. Serotonin and tyrosine hydroxylase immunoreactive axons were more extensively present in mouse spinal cords treated with ARSB and ChaseABC, and the immunoreactive axons penetrated further beyond the injury site in ARSB or ChaseABC treated mice than in control mice. These results indicate that mammalian ARSB improves functional recovery after CNS injury. The structural/molecular mechanisms underlying the observed functional improvement remain to be elucidated.

## Introduction

Unlike fish and many non-mammalian vertebrates, mammals show a very limited capacity for regeneration after acute and chronic injury of the adult central nervous system [1]–[4]. Several cellular and molecular mechanisms may underlie this limitation including a paucity of conducive, and an abundance of inhibitory contributions to the damaged tissue to heal and to renew functions operant before injury. Among molecules identified in many studies to inhibit growth are members of the chondroitin sulfate proteoglycan (CSPG) family, which are up-regulated in expression in the injured parts of the central nervous system [5]–[7]. CSPGs are mostly localized in the extracellular matrix and are major formative determinants during nervous system development [8]–[11]. Their importance in synaptic functions and plasticity in the adult is increasingly becoming recognized, particularly regarding the striking structures of perineuronal nets whose contributions to interneuronal activity have been mainly unresolved, but are thought to limit plasticity [12]–[14]. Production of CSPGs increases in injured spinal cords in astrogliotic tissues around the lesion site [15]–[17]. The glial scar resulting from astrogliosis is considered a mechanical and molecular barrier to growing axons from supraspinal nuclei in brainstem and somatosensory cortex [18], [19].

Glycosaminoglycan (GAG) chains mediate the inhibitory activities of CSPGs. Sulfate additions appear to be crucial determinants of inhibitory activities of GAG chains. Four different types of chondroitin sulfate (CS) sulfation (4*-O-*sulfation, C4S; 6*-O-*sulfation, C6S; 2,6*-O-*sulfation, C2, 6S; 4,6*-O-*sulfation, C4, 6S) inhibit neurite outgrowth and affect intracellular signaling. C4S demonstrated to be the major contributor of inhibiting axon outgrowth at the spinal cord lesion site [20], while C6S, also presented to be an inhibitory determinant in CNS injury [21], by inhibiting cortical neuronal outgrowth, which was blocked by treatment with C6S binding peptides [22], [23]. Interestingly, C6S may paradoxically play a permissive role for neuronal regeneration. C6S-sulfotransferase knockout mice have diminished nigrostriatal axonal regeneration, while peripheral axons are unaffected [24]. In agreement with this observation, up-regulation of C6S-sulfotransferase-1 facilitates Schwann cell migration in the injured sciatic nerve [25]. Recent studies show that C4, 6S has an inhibitory role in injured cortex where it (along with C2S, C6S, but not C4S) was up-regulated in peri-infarct gliotic tissue compared to uninjured cortex. In addition, C4, 6S strongly inhibits dorsal root ganglion neurite growth [26], while targeted knockdown of C4, 6S-sulfotransferase, using shRNA, mitigated CSPG-mediated inhibition [27]. CSPGs have seemingly multi-functional roles but despite a few instances of growth permissiveness, the above-mentioned CSPGs have primarily been an obstacle to axon outgrowth.

The bacterial enzyme ChaseABC removes GAG chains of CSPGs [17], [28], [29]. The therapeutic potential of this enzyme has been repeatedly documented to promote locomotor recovery and axonal regrowth in different types of spinal cord injury paradigms in rodents [17], [30], [31], not only when applied by itself, but also in combination with other conducive agents, such as neurotrophins, stem cells, and the neural adhesion molecule L1 [32]–[34]. Furthermore, this enzyme is not only beneficial in acute stage of CNS injury, but in chronic spinal cord injury paradigms as well [3], [35].

Treatment of spinal cord injured patients with ChaseABC has not yet reached clinical trials, possibly due in part to the potential immunogenicity of this bacterial protein and its chemical instability in injured tissues. Injured CNS develops a local acidic environment, of approximately pH 6.8 [36]–[38], while ChaseABC has an activity optimum pH of 8 [39]–[41]. In addition, ChaseABC loses activity at the mammalian body temperature of 37°C [40], [42], and necessitates modification to increase its thermostability to remain active at that temperature [43]. Given the above characteristics of ChaseABC, we decided to probe the therapeutic potential of a less immunogenic and more chemically stable mammalian enzyme that removes sulfates and in particular, the C4S moieties from CSPGs in spinal cord injury. ARSB (N-acetylgalactosamine 4-sulfatase) lends itself to such analysis, since it has an acidic pH optimum [44] and is approved to treat patients with mucopolysaccharidosis type VI [45]–[47]. It functions in lysosomal removal of C4S groups from chondroitin and dermatan sulfate; these C4 sulfate groups, in extracellular CS, were shown to be the major structural determinant for axonal growth inhibition [20]. Recent findings report that ARSB is also localized in the extracellular matrix [48]. Here, we report that a one-time injection of human ARSB into the acute lesion site and in the close vicinity of this site in a mouse compression model of spinal cord injury leads to improved locomotor activity and re-growth/sprouting of descending axons. These observations encourage the expectation that human ARSB will be an added benefit to other modes of treatment for spinal cord injury and other injuries of the central nervous system in humans.

## Results

### 4-O-sulfated GAG Abundance after Compression Injury and Injection of ARSB

**Figure 1:**
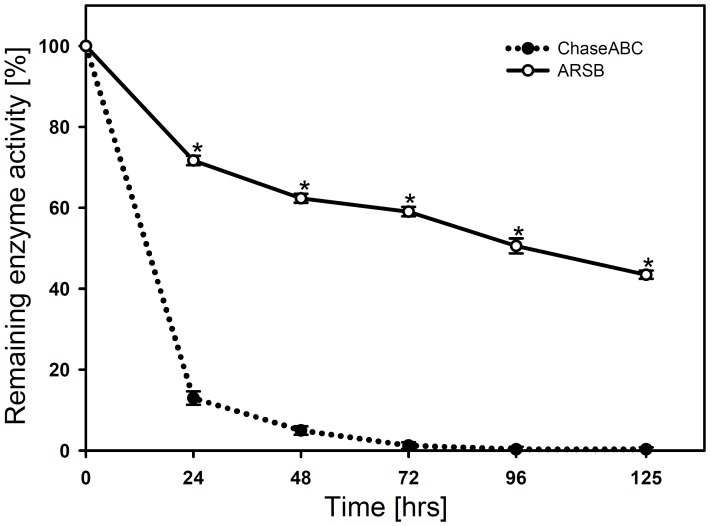
Thermostability of ARSB and ChaseABC. Stock solutions of each enzyme were incubated at 37°C/pH 6.8 for up to 125 hours. At the indicated time points aliquots were diluted into pre-warmed assay buffer (pH 8.0 for ChaseABC, pH 5.6 for ARSB) and subjected to activity determination (see Methods). Asterisks indicate significant differences between the groups *p<0.001 as assessed by t-Test. Data represent means ± SEM (n = 4).

**Figure 2:**
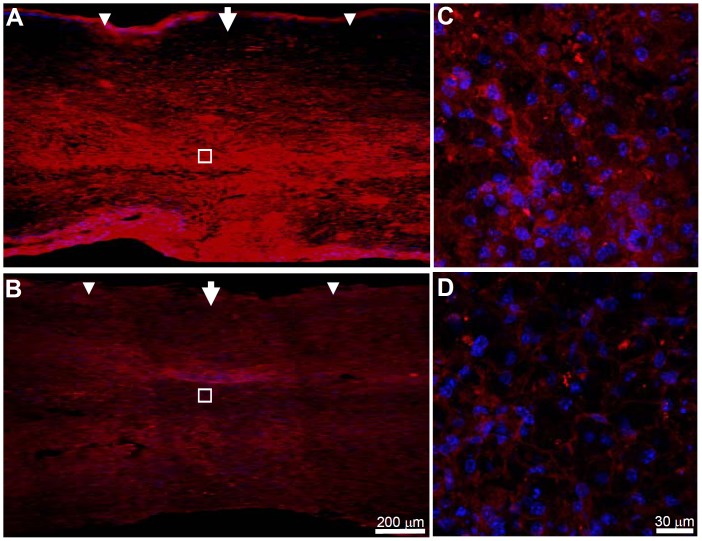
Chondroitin 4*-O-*sulfate (C4S) immunoreactivity is reduced at five days after ARSB injection after moderate compression-injury. Immediately after spinal cord injury, one µl of ARSB (10 U/ml) was injected at the injury site (arrow) and 0.5 mm rostral and caudal to this site (arrowheads). After 5 days, the mice were perfused, and sagittal spinal cord sections were analyzed by immunofluorescence using an antibody specific for C4S. C4S immunoreactivity is higher at the injury site in the buffer treated control mice (**A**) versus ARSB treated mice (**B**). (**C**) and (**D**) are higher magnifications of the insets in (**A**) and (**B**), respectively. Diamidino-phenylindole (DAPI, blue) was used for nuclear staining and merged with C4S immunoreactivity (**C,D**). Arrows and arrowheads indicate the injury site and injection sites, respectively.

We assessed the thermostability of ARSB in comparison to ChaseABC at 37°C and at the relevant pH range of 6.0 to 6.8. Under these conditions ARSB retained half of its activity even after 5 days, while ChaseABC at pH 6.8 lost approximately 90% of its activity within 24 hours, and was fully inactive after 3 days (**Fig. 1**); at pH 6.0 ChaseABC was completely inactived already after 24 hours (data not shown). We then investigated whether human ARSB would remove C4S immunoreactivity in sagittal sections of injured mouse spinal cord after directly injecting the enzyme after moderate compression injury into the spinal cord tissue at the lesion site and 0.5 mm rostral and caudal to the site. Five days after injection, immunofluorescent staining was performed on formaldehyde fixed tissue in sagittal sections, serially spaced 400 µm apart, comprising the lesion site and several micrometers (∼100 µm) in its vicinity (**Fig. 2** **, Fig. S1**). An antibody specific for detection of C4S was used to show that spinal cord tissue treated with ARSB showed considerably less immunoreactivity than spinal cords treated with vehicle only (compare **Figs. 2a,c** with **Figs. 2b,d**). These observations indicate that after injection of ARSB, C4S immunoreactivity is reduced in the extracellular space under the physiological conditions of the host tissue, either as a direct or indirect consequence of this enzyme activity.

### Sulfated GAG Levels after Injection of ARSB and ChaseABC

**Figure 3:**
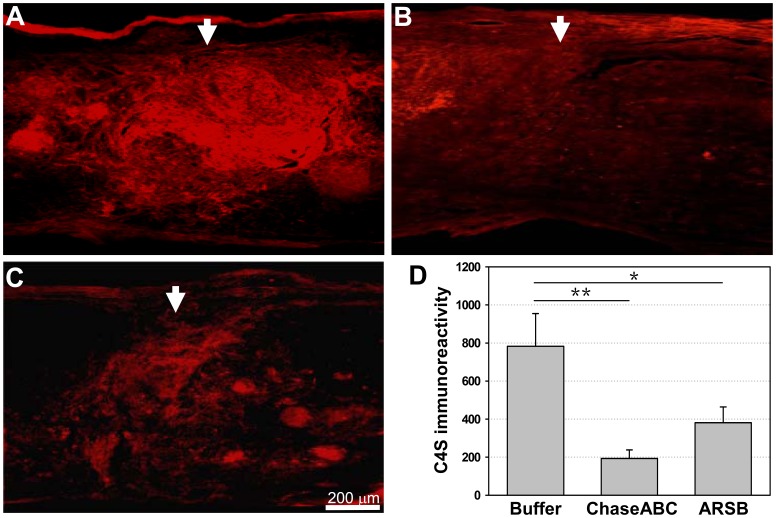
C4S immunoreactivity is reduced at 9 weeks after enzyme injection in severe compression injury. Immediately after spinal cord injury**,** one µl ChaseABC (10 U/ml), ARSB (10 U/ml) or buffer was injected at the injury site and 0.5 mm rostral and caudal to this site. After 9 weeks, the mice were perfused, and sagittal spinal cord sections were analyzed by immunofluorescence using an antibody specific for C4S. C4S immunoreactivity is more intense at the injury site in the buffer treated control mice (**A**) versus the ChaseABC (**B**) and ARSB (**C**) treated mice. Immunoreactivities of the entire images were quantified above threshold using Image J software (**D**). Mean fluorescence intensities of the area at 0.4 mm equidistant rostral and caudal to the center of the injury site show significantly less C4S immunoreactivity in ChaseABC and ARSB treated mice versus buffer treated control mice. Reduction of immunoreactivity is not significantly different between applications of ChaseABC versus ARSB. Arrows indicate the injury site. Asterisks indicate significant differences between the groups *p<0.05 and **p<0.01 as assessed by one-way ANOVA followed by Tukey’s *post-hoc* analysis. Data represent means ± SEM (n = 4 mice).

**Figure 4:**
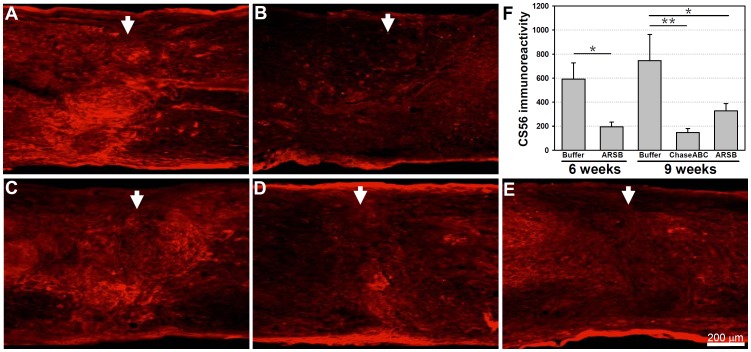
C4S and C6S immunoreactivities are reduced at 6 and 9 weeks after ARSB and ChaseABC treatment. Immediately after moderate (**A,B**) and severe (**C–E**) spinal cord injury, one µl ChaseABC (10 U/ml), ARSB (10 U/ml) or buffer was injected at the injury site and 0.5 mm rostral and caudal to this site. After 6 (**A,B**) and 9 weeks (**C–E**), the mice were perfused, and sagittal spinal cord sections were analyzed by immunofluorescence using the CS56 antibody reacting with C4S and C6S. Immunoreactivity is more intense at the injury site in the buffer treated control mice (**A,C**) versus the ChaseABC (**D**) and ARSB (**B,E**) treated mice. Immunoreactivities of the entire images were quantified above threshold using Image J software (**F**). Mean fluorescence intensities of the area at 0.4 mm equidistant rostral and caudal to the center of the injury site show significantly less expression of CSs in ChaseABC and ARSB treated mice versus buffer treated control mice. Reduction of immunoreactivity is not significantly different between applications of ChaseABC versus ARSB. Arrows indicate the injury site. Asterisks indicate significant differences between the groups *p<0.05 (ARSB) and **p<0.01 (ChaseABC) as assessed by one-way ANOVA followed by Tukey’s *post-hoc* analysis. Data represent means ± SEM, (n = 3 mice).

We then compared the efficacy of C4S removal 9 weeks after injury between human ARSB and the bacterial enzyme ChaseABC treated mice, with equal enzymatic units injected per mouse, using the same injection protocol as for the immunofluorescent assessment five days after the injection of ARSB. In addition to the C4S-specific antibody, antibody CS56 (specific for both C4S and C6S) was used in parallel for immunofluorescent staining on consecutive spinal cord sagittal sections, and quantified with Image J software (**Figs. 3**, **4**). In comparison to the tissue harvested from the vehicle only treated spinal cords, both enzymes removed C4S immunoreactivity significantly when the same quantities of enzyme units were injected, but ChaseABC reduced C4S immunoreactivity more effectively than ARSB. (For immunofluorescent images of sagittal sections, see **Figs. 3a–c**; and see **Fig. 3d** for the quantitative assessment of immunofluorescence intensity of each entire image in **Figs. 3a–c**). A similar result was obtained when immunofluorescent staining was performed with the GAG sulfate antibody CS56, which recognizes both C4S and C6S; both enzymes removed immunoreactivity significantly, but ChaseABC reduced immunoreactivity more efficiently than ARSB when assessed at 6 and 9 weeks after application (**Fig. 4a–f**).

### Assessment of Locomotor Function

**Figure 5:**
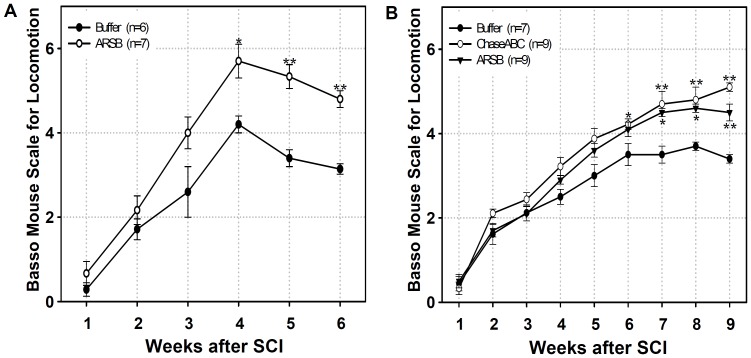
Functional recovery of ChaseABC or ARSB treated mice after moderate (A) and severe (B) compression. One µl ChaseABC (10 U/ml), ARSB (10 U/ml) or buffer was injected at the injury site and 0.5 mm rostral and caudal to this site. The Basso Mouse Scale for analysis of locomotor activity was used to score functional recovery up to 6 (**A**) and 9 (**B**) weeks after moderate and severe spinal cord injury (SCI), respectively. Asterisks indicate significant differences between the enzyme treated groups versus the buffer treated control group at the same time points (*p<0.05, **p<0.01). No significant difference is detectable between the ChaseABC and ARSB treated groups by one-way ANOVA followed by Tukey’s *post*-*hoc* analysis. Data represent means ± SEM. Number of mice are indicated in brackets.

Two sets of experiments were performed to assess locomotor recovery after spinal cord injury and treatment with ARSB. The first experiment was designed to test whether injection of ARSB would enhance locomotor recovery by 6 weeks, compared to the control, in a moderate compression injury paradigm. Since this trial yielded promising results, we performed a second set of experiments using a severe compression injury paradigm including, for comparison, the bacterial enzyme ChaseABC. To test whether reduction of CS immunoreactivity at and around the lesion site would lead to better functional recovery, we next tested locomotor parameters weekly for 6 and 9 weeks after injury by comparing the vehicle only treated control mice to the enzyme treated mice (**Fig. 5**). The vehicle only treated mice showed spontaneous locomotor recovery within the expected range when scored by the BMS. Both ARSB and ChaseABC improved locomotor recovery, which became significantly different at 6 weeks after enzyme injection for ChaseABC, and at 7 weeks after enzyme injection for ARSB when compared to the control. It is noteworthy that no significant differences could be detected in the extent of locomotor recovery between ARSB and ChaseABC treated mice at the later time points after enzyme injection. In daily inspections of the injured mice, we detected no signs of autophagia or other indications of negative reactions to enzyme treatments.

### Assessment of Glial Scar Size

**Figure 6:**
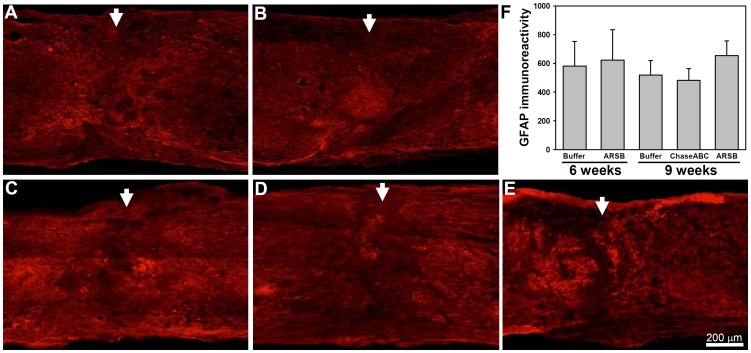
GFAP immunoreactivity is not different at 6 or 9 weeks after enzyme treatment. One µl of the ChaseABC (10 U/ml), ARSB (10 U/ml) or buffer was injected at the injury site and 0.5 mm rostral and caudal to this site after moderate and severe compression injury. After 6 (**A,B**) and 9 weeks (**C–E**), the mice were perfused, and sagittal spinal cord sections were analyzed by immunofluorescence for glial fibrillary acidic protein (GFAP). Photomicrographs show GFAP immunoreactivity at the injury site in the buffer treated control mice (**A,C**) versus ChaseABC (**D**) and ARSB (**B,E**) treated mice. Immunoreactive areas were quantified above threshold using Image J software (**F**). Mean fluorescence intensities of the area at 0.4 mm equidistant rostral and caudal to the center of the injury site are similar in all groups. Arrows indicate the injury site. One-way ANOVA followed by Tukey’s *post-hoc* analysis indicates no difference between the groups (p>0.9). Data represent means ± SEM (n = 3 mice).

As the glial scar is considered to be a mechanical and molecular barrier to axonal re-growth/sprouting, the size of the glial scar at the lesion site was investigated using an antibody against GFAP, which detects normal and activated astrocytes. In sagittal sections from spinal cords 6 and 9 weeks after enzyme injections, no differences were detectable between the three groups (**Fig. 6**).

### Assessment of Serotonin Expressing Axons

**Figure 7:**
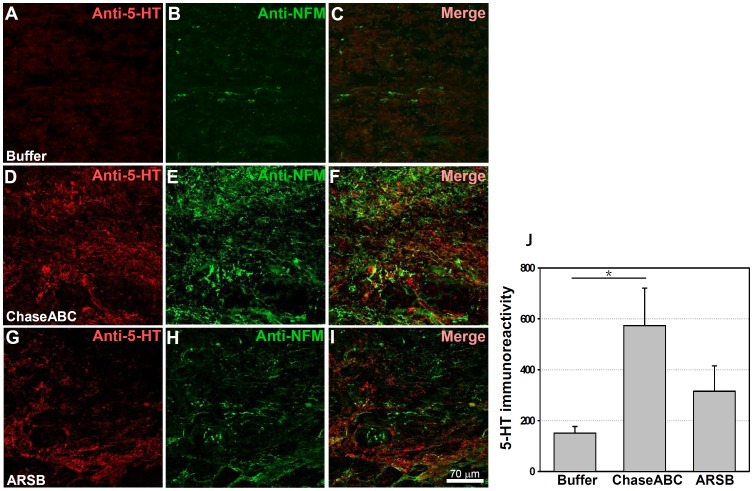
5-HT immunoreactive axons are more abundant 9 weeks after enzyme treatment than in control mice. One µl ChaseABC (10 U/ml), ARSB (10 U/ml) or buffer was injected at the injury site and 0.5 mm rostral and caudal to this site in mice with severe compression injury. After 9 weeks, the mice were perfused, and sagittal spinal cord sections were analyzed by immunofluorescence. Double immunostaining for serotonin (5-HT) and neurofilament-M (NF-M) shows higher immunoreactivities caudal injury site in the ChaseABC (**D,E,F**) and ARSB (**G,H,I**) treated mice versus buffer treated control mice (**A,B,C**). (**A,D,G**) Immunostainings for 5-HT and (**B,E,H**) NF-M, and (**C,F,I**) merged for 5-HT with NF-M. 5-HT immunoreactive axons are seen beyond the injury site in the ChaseABC and ARSB injected mice. Immunoreactive areas were quantified above threshold using Image J software (**J**). Mean fluorescence intensity of the area between the injury site and 1 mm caudal to it is significantly higher in ChaseABC treated versus buffer treated control mice. Asterisk indicates significant differences between the groups *p<0.05 by one-way ANOVA followed by Tukey’s *post-hoc* analysis. Data represent means ± SEM, (n = 5 mice).

Axons projecting from the brainstem are decisive contributors to locomotor functions when lesions are performed at the thoracic trunk level of the spinal cord. The immunoreactivities and lengths of serotonergic (5-HT) axons extending beyond the caudal margin of the injury site were measured by double immunostaining with 5-HT and neuronal intermediate filament-M (NF-M), 9 weeks after enzyme injection. Mice treated with ChaseABC tended to have more axons extending beyond the lesion site (**Fig. 7**). The difference between the groups of mice that had received either enzyme was not significant, but when compared to the vehicle control, the differences were significant. Mean fluorescent intensity of 5-HT immunoreactive axons penetrating into the caudal margin of the injury site was also higher for the ChaseABC treated mice than for the ARSB (**Fig.** **7j**).

### Assessment of Tyrosine Hydroxylase Expressing Axons

**Figure 8:**
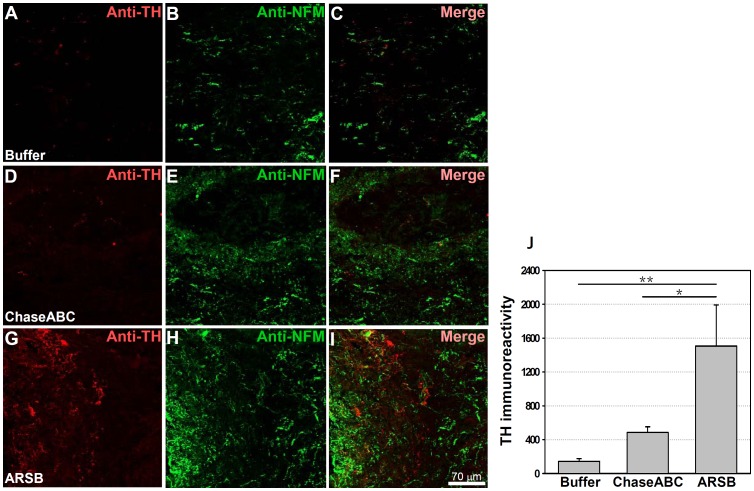
Tyrosine hydroxylase (TH) immunoreactive axons are more abundant 9 weeks after enzyme treatment than in control mice. One µl ChaseABC (10 U/ml), ARSB (10 U/ml) or buffer was injected at the injury site and 0.5 mm rostral and caudal of this site in mice with severe compression injury. After 9 weeks, the mice were perfused, and sagittal spinal cord sections were analyzed by immunofluorescence. Double immunostaining for TH and neurofilament-M (NF-M) shows higher immunoreactivities caudal to the injury site in the ChaseABC (**D,E,F**) and ARSB (**G,H,I**) treated mice versus the buffer treated control mice (**A,B,C**). (**A,D,G**) Immunostainings for TH and (**B,E,H**) NF-M, and (**C,F,I**) merged for TH with NF-M. Immunoreactive areas were quantified above threshold using Image J software (**J**). Mean fluorescence intensity of the area between the injury site and 1 mm caudal to it shows significantly higher immunofluorescence intensity in ARSB versus buffer treated control and ChaseABC treated mice. Asterisks indicate significant differences between the groups *p<0.05 and **p<0.01 as assessed by one-way ANOVA followed by Tukey’s *post-hoc* analysis. Data represent means ± SEM, (n = 4 mice).

A different picture emerged when the tyrosine hydroxylase (TH) immunoreactive axons, which also originate from the brainstem, were analyzed (**Fig. 8**) by double immunofluorescent staining with NF-M. Application of either enzyme enhanced the lengths and numbers of TH immunoreactive axons beyond the lesion site 9 weeks after enzyme injection when compared to the control. The difference between the groups of mice that had received either enzyme was significant from one another as well as the vehicle control. Both enzymes showed more TH immunoreactive axons in the injury of caudal area when compared to the control when double immunostained (**Fig. 8c** versus **Fig. 8f** and **Fig. 8i**). Treatment with ARSB led to significantly more TH immunoreactive axons in and beyond the caudal margin of the injury site than treatment with ChaseABC (**Fig. 8j** **)**.

### Assessment of Microglia/Macrophages

**Figure 9:**
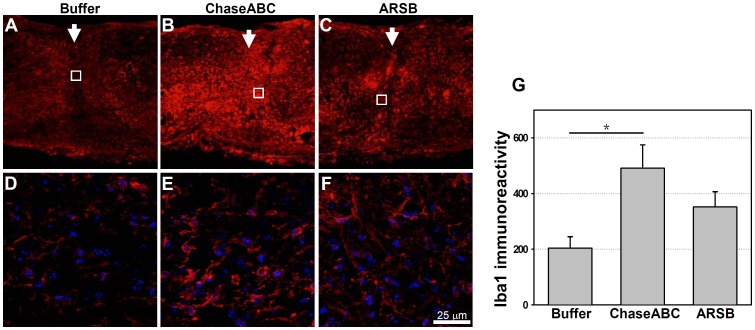
Iba1 immunoreactive microglia/macrophages are more abundant 9 weeks after ChaseABC treatment than in control mice. One µl ChaseABC (10 U/ml), ARSB (10 U/ml) or buffer was injected at the injury site and 0.5 mm rostral and caudal to this site in mice after severe compression. After 9 weeks, the mice were perfused, and sagittal spinal cord sections were analyzed by immunofluorescence. Immunoreactivity for Iba1 is less intense at the injury site in the buffer treated control mice (**A**) versus ChaseABC (**B**) or ARSB (**C**) treated mice. (**D**) (**E**) and (**F**) are higher magnifications of the insets in (**A**) (**B**) and (**C**), respectively, showing more microglia/macrophages with activated, amoeboid morphology in the area between the injury site and 1 mm caudal to it of mice treated with ChaseABC (**E**) or ARSB (**F**) versus the buffer treated control mice (**D**). Immunoreactive areas were quantified above threshold using Image J software (**G**). Mean fluorescence intensities at 0.4 mm equidistant rostral and caudal to the center of the injury site shows significantly higher immunoreactivity for Iba1 in ChaseABC treated mice versus buffer treated control mice. Arrows indicate the injury site. Diamidino-phenylindole (DAPI, blue) was used for nuclear staining and merged with Iba1 immunostaining (**D,E,F**). Asterisk indicates a significant difference between the groups *p<0.05 as assessed by one-way ANOVA followed by Tukey’s *post-hoc* analysis. Data represent means ± SEM, (n = 3 mice).

Since the immune response is considered to be a major obstacle to the use of the bacterial form of the enzyme, the presence of activated microglia/macrophages was investigated using an antibody to the ionized calcium binding adaptor molecule 1 (Iba1). Iba1 positive microglia/macrophages persisted at the injury site in the ChaseABC, but not the ARSB treated group, the latter being more similar to the buffer only control group (**Figs. 9a–c**). At the epicenter of the injury site, Iba1 positive cells can be observed in all treatment groups with significantly more microglia/macrophages detected after application of ChaseABC compared to the control or ARSB groups (**Fig. 9g**). Iba1 positive cells in all treatment groups displayed an “activated” morphology indicated by short, thick processes. However, the ChaseABC treated group had more Iba1 positive cells with amoeboid morphology than the buffer control and ARSB treated animals (**Figs.** **9d–f**).

## Discussion

Since ARSB would have considerable advantages over ChaseABC for therapeutic use in humans, we tested its performance in a rodent model of spinal cord injury. Indeed, we have demonstrated here that the human enzyme ARSB is more thermostable than the bacterial enzyme ChaseABC, and a one-time injection of ARSB immediately after injury enhanced locomotor recovery after both six weeks moderate and 9 weeks severe compression injury compared with buffer injected control mice. Immunofluorescent analysis suggests that functional effects are mediated by a reduction of C4S expression. Locomotor recovery was similar between mice injected with equal units of ARSB or ChaseABC, and both groups showed enhanced recovery relative to control mice. It is noteworthy that in both sets of experiments, serotonergic (5-HT) and tyrosine hydroxylase (TH) immunoreactive axons were more abundant beyond the injury of caudal area in the groups treated with ARSB and ChaseABC than in the control group. In addition, Iba1 immunoreactivity was significantly higher in the ChaseABC group than in the buffer control and ARSB treated groups at 9 weeks.

### Improved Functional Recovery

Many investigators have documented that intraparenchymal ChaseABC injection enhances neurite outgrowth and improves locomotor recovery from the spinal cord injury in rodent paradigms [30]–[35], [49]–[51]. In our study, we showed that ARSB enhanced locomotor function. Depending on the severity of the lesion, the beneficial effects of enzyme treated group were observed 4 or 7 weeks after moderate and severe cord compression injury, respectively. The first set of experiments treating moderate compression followed by injection of ARSB, revealed significantly better locomotor recovery than control mice at 4 weeks after injury. After severe injury and treatment with equal units of either bacterial ChaseABC or human enzyme ARSB, animals showed functional improvement after approximately 6 weeks, with no significant difference between the enzyme-treated groups. However, both enzyme-treated groups showed significant improvement over buffer-treated control mice. This delay over the first 3 weeks after severe compression injury can be attributed to injury severity, as control mice did not surpass a BMS score of 4. ChaseABC treated mice tended to show slightly, although not significantly better improvement relative to ARSB. This result may be because ChaseABC degrades GAG chain at the C4S and C6S, whereas ARSB degrades C4S without change of GAG chain. If confirmed in further experiments, this observation would raise the question of whether both C4S and C6S contribute to inhibition of neuritogenesis and functional remodeling of perineuronal nets. Like C4S, C6S increases in the spinal cord after injury [20] and inhibits axonal regeneration in cortical brain lesions [21]. However, other studies reported that C4S is neither up-regulated after cortical injury nor inhibitory to dorsal root ganglion axon outgrowth *in vitro*, and suggested C4, 6S (CS-E) is a potentially inhibitory molecule [26]. C6S binding peptides, obtained from a peptide phage display library, can block C6S and enhance cortical neurite outgrowth [22], [23], suggesting an inhibitory role of C6S for cortical neurons. It is, however, also conceivable that application of ARSB is not as efficient as ChaseABC in changing GAG chain composition and/or that removal of GAG chain of C6S by ChaseABC may allow better access to the C4S specific antibody, thus yielding higher fluorescence intensity levels by immunohistochemistry using specific C4S antibody LY111 [52].

The chemical determination of CS chain structural changes due to ARSB application, in GAGs retrieved from live tissue, is not feasible with presently available methods of CS compositional analysis, since even with the most refined methods practiced in highly specialized laboratories [20], [53] the tissue amounts needed would be overwhelming. Recently developed methods for heparan sulfate compositional analysis [54] await adaptation to CS analysis. Even if this method of analysis was available, interpreting the results would be problematic since ARSB, unlike ChaseABC, is not a lyase (chondroitinase), but acts as an exosulfatase [55], [56] generating non-reducing end structures that may initiate unknown molecular interactions and signaling events. In fact the sulfation status of CS has been described to trigger several signaling pathways including pleiotrophin and HGF signaling during neuritogenesis [57]. It is noteworthy in this context to mention that addition of chondro-4-sulfatase, which like ARSB preferentially acts as an exo-4-sulfatase, to cultures of neurons in spot assays abolished the axon-repellent action of CS [20]. Analysis of CS structural changes due to ARSB is desirable, but the difficulty in interpreting any results obtained from such analysis is that application of ARSB into the injured tissue most probably leads to additional cellular and molecular consequences that are likely to be indirectly caused by ARSB activity at any time point after injury. In addition, such consequences may involve regulation (expression, release or activation) of endogenous biosynthetic or degradative enzymes as part of feedback mechanisms, in analogy to biosynthetic heparan sulfate remodeling, which has been described as a consequence of extracellular desulfation [58]. It should further be taken into consideration that presently, from the combined reports available, it is not yet clearly resolved which CS sulfation patterns are regeneration prohibitive or promoting. Thus, the aim of the present study was not to characterize the structure of the GAG chains resulting from the action of ARSB, but to determine the possibility to use a clinically approved enzyme in a central nervous system injury animal model.

Some relationships between CSPGs, ChaseABC, axonal regeneration, and functional recovery are well documented, but the mechanisms of different CSPGs are not well understood. A recent study showed that CSPG production starts after breakdown of the blood brain barrier, mediated by the TGF-β/Smad pathway triggered by blood fibrinogen [59] and various Smad isoforms differentially regulate different CSPGs [60]. The build up of CSPG around the injury site serves as a growth-inhibiting signal for axons, and is also required for infiltrating immune cells to promote healing processes. This was demonstrated by CSPGs interacting with monocytes to stimulate their differentiation to M2 macrophages (anti-inflammatory) that secrete interleukin 10 (IL-10) and matrix metalloproteinase 13 (MMP13) to dissolve CSPGs, and attempt to resolve inflammation [61], [62]. Thus, it is clear that CSPGs have differential effects on multiple cell types, and future experiments examining these effects in greater detail should be enlightening.

### Glial Scar Formation after Injury

Many investigators have focused on formation of the “glial scar” after spinal cord injury. The “scar” is thought to be both a molecular barrier, via the expression of inhibitory molecules, and physical barrier that prevents axonal re-growth. It is also thought to regulate interactions between central nervous parenchyma and the blood brain barrier, preventing spread of cellular damage and minimizing infection [63]. Previous studies [34], [49] have shown that glial scar size did not differ between experimental groups at 6 and 9 weeks after both moderate and severe spinal cord injury. However, quantification of GFAP immunoreactivity in the present study indicated increased astrogliosis after injury, as also reported previously [19], [64].

### Serotonin- and Tyrosine Hydroxylase-immunoreactive Axons

Serotonergic (5-HT) and tyrosine hydroxylase (TH) immunoreactive neurons are able to regenerate after spinal cord injury (for more recent publications, see papers by Alilain, et al. and Hawthorne, et al.) [65], [66]. When 5-HT positive neurons were transplanted into an injured rodent spinal cord, they elongated and integrated into the CNS parenchyma leading to functional recovery [63]. We show here that both types of axons do indeed penetrate into and extend beyond the injury site. However, whereas TH immunoreactive axons extend beyond the lesion site slightly better after application of ARSB than after application of ChaseABC, application of ChaseABC resulted in considerably longer 5-HT axonal lengths 9 weeks after severe compression injury than after application of ARSB. We interpret these results as indicating that 5-HT and TH immunoreactive axons respond differently to the different types of enzyme action on CSs. Yet, in the overall assessment of locomotor function this difference does not appear to play a significant role.

### Microglia/Macrophage Response after Enzyme Treatment

A robust and persistent inflammatory response occurs in the spinal cord after injury, with microglia/macrophages playing an important role in pathology and repair. These cells generally exacerbate injury and lead to poor recovery from CNS injury [67], [68], but some investigators [64] have found otherwise. In the present study, the ChaseABC treated group differed significantly from vehicle controls and the ARSB treated groups when assayed for Iba1 positive microglia/macrophages (see **Fig. 9**). Interestingly, the ChaseABC group showed a greater number of macrophages with amoeboid morphology, suggesting that there is more active phagocytosis at 9 weeks after injury and treatment with ChaseABC, than with ARSB or control buffer. This difference is possibly due to eliciting a strong microglia/macrophage mediated immune response to the bacterial enzyme or resulting from GAG chain degradation from the extracellular environment of perineuronal net.

Major advantages of ARSB include its long-term stability at physiological temperature and increased enzymatic activity at acidic pH, which are clearly superior to the values for ChaseABC (**Fig. 1**). As local tissue acidification due to CNS injury stimulates ARSB activity, but reduces ChaseABC activity, ARSB is well suited for use in mammals. An additional and equally important advantage of ARSB over ChaseABC is its reduced immunogenicity in the mammalian host. Lastly, we demonstrate that a one-time injection of ARSB is sufficient to promote functional recovery. Taken together, these advantages make ARSB a particularly attractive candidate for treating not only spinal cord injury. In order to unravel the cascade of events finally leading to improved recovery, CSPG degradation as the proposed underlying mechanism will need to be confirmed in future experiments.

## Materials and Methods

### Production, Purification and Determination of Enzymatic Activity of Human ARSB and Bacterial ChaseABC

ARSB was expressed and purified to homogeneity as described [69]. The enzyme preparation used had a specific activity of 34 U/mg at a protein concentration of 0.35 mg/ml. We determined enzyme activity by using the artificial substrate pNCS (p-nitrocatechol sulfate dipotassium salt; Sigma, St. Louis, MO) following reported protocols [70], [71]. Protease-free ChaseABC (Seikagaku, Tokyo, Japan) from Proteus vulgaris was assayed after a modified method [29]. The reaction buffer consisted of 40 mM Tris, 40 mM sodium acetate and 0.01% (w/v) BSA at a pH of 8.0. Briefly, 20 µl of the enzyme solution were added to 100 µl of pre-warmed chondroitin sulfate substrate (Sigma, from shark cartilage, dissolved at a concentration of 4 mg/ml in reaction buffer) in a Greiner UV-Star microplate. The increasing absorbance at 232 nm was assayed continuously for 25 min at 37°C, thus allowing activity calculation from the slope of the time course (absorbance/time). The temperature stability of ChaseABC and human ARSB was assessed at pH 6.0 (in 20 mM MES, 150 mM NaCl) and at pH 6.8 (20 mM MOPS, 150 mM NaCl) by incubating these solutions at 37°C for different times (up to five days, as given in the figure), before measuring enzyme activity at pH 5.5 (ARSB) or pH 8.0 (ChaseABC), as described above.

### Animals

Eight-week-old C57BL/6 female mice were purchased from the Charles River Laboratories (Wilmington, MA). All experimental procedures were approved by the Animal Care and Facilities Committee of Rutgers, The State University of New Jersey. The mice were anesthetized with Ketamine-Xylazine (see details below) and subjected to spinal cord compression after a laminectomy. After spinal cord injury, the animals were maintained in the core animal facility at the Division of Life Science and the W.M. Keck Center for Collaborative Neuroscience. After surgery, the mice were kept on a warm mat (35°C) for several hours to prevent hypothermia and were thereafter singly housed in a temperature- and humidity-controlled room with water and standard food provided *ad libitum*. The bladders of the animals were manually voided once or twice daily if the bladder is manually palpable. The animals were euthanized under anesthesia and transcardially perfused with 4% paraformaldehyde to fix the tissues for histological and other analyses.

We performed two sets of experiments. In the first set, 7 mice received moderate compression injury and one intraspinal injection of human ARSB in Tris-buffered saline, pH 7.3 (TBS), the optimal buffer for enzyme stability. An additional 6 mice received TBS as vehicle control. In the second set, 9 mice received ARSB, the bacterial enzyme ChaseABC (Seikagaku, Tokyo, Japan), and 7 mice received TBS vehicle only as control. The mice were assessed for locomotor activity for 6 weeks in the first set and 9 weeks in the second set.

### Surgery and Intraspinal Injection

After anaesthetizing the mice with an intraperitoneal injection of Ketamine (160 mg/kg (Butler Schein Animal Health, Chicago, IL) and Xylazine (24 mg/kg, Butler Schein Animal Health, Chicago, IL), 0.1 ml of 0.125% Bupivacaine (Hospira, Inc., Lake Forest, IL) was injected around the incision site to provide local anesthesia. A three cm skin incision along the median line on the back of the animals was made. Laminectomy was performed with Mouse Laminectomy Forceps (Fine Science Tools, Heidelberg, Germany) at the T7–T9 level, followed by a mechanically controlled compression injury using a mouse spinal cord compression device [72]–[74]. This device contains a pair of watchmaker forceps sterilized by a bead sterilizer, mounted onto a metal block attached to a stereotaxic frame and automated with regard to the force and time of compression by an electromagnetic device. The spinal cord was compressed for 0.5 seconds for the moderate compression injury and 1 second for the severe compression injury by a timed current through the electromagnetic drive. Injections were performed immediately after compression injury by inserting a 33-gauge needle connected to a 5 µl Hamilton syringe (Hamilton, Reno, NV) in a stereotactic micromanipulator (Narishige, New York, NY). ChaseABC, or ARSB (10 units/ml), or the control TBS solution was injected 1 mm deep into the cord midline (1 µl at the lesion center and 1 µl each at sites 0.5 mm rostral and caudal to the lesion center), with each injection lasting for 7 min. After injection, the skin was closed with wound clips.

### Locomotor Assessment

We assessed locomotor function by the BMS score [74], [75] one week before and every week after injury. For assessment of the BMS, the mice were allowed to move in an open field, 1 meter in diameter, for 5 min. The hindlimb movements were observed and scored according to the BMS scale by two expert and independent observers, blinded to the treatment. Statistical analyses were performed using repeated measures analysis of variance (one-way ANOVA), followed by Tukey’s *post-hoc* test. The p values <0.05 were considered statistically significant.

### Immunohistology

Animals were anaesthetized with an intraperitoneal injection of Ketamine and Xylazine followed by transcardial perfusion with 4% paraformaldehyde in phosphate-buffered saline, pH 7.3 (PBS), following vascular washout with PBS. After removing the spinal cords and cryoprotecting them by incubation in 20% sucrose solution overnight at 4°C, they were frozen and cut into 20-µm-thick serial, sagittal sections, including tissue rostral and caudal to the lesion site. The sections were mounted on microscope slides and saved at −80°C for immunofluorescent detection. The slides with sections of equivalent distance from the lesion center of each group were thawed to room temperature and then washed three times and blocked with 10% goat serum for 2 h at room temperature. Slides were incubated overnight at 4°C with mouse monoclonal anti-chondroitin sulfate antibody (CS56) (1∶200, cat# C8035, Sigma-Aldrich, St. Louis, MO), mouse monoclonal anti-chondroitin-4-sulfate antibody (C4S) (1∶100, cat# 270421, Seikagaku, Tokyo, Japan), and rabbit polyclonal antibodies to glial fibrillary acidic protein (GFAP) (1∶200, cat# G4546, Sigma-Aldrich, St. Louis, MO), tyrosine hydroxylase (TH) (1∶500, cat# AB152, Millipore, Temecula, CA), serotonin (5-HT) (1∶400, cat# 10385, Abcam, Cambridge, MA) to Iba1 (1∶800, Wako, Osaka, Japan) and to neurofilament-M (NF-M) (1∶1000, cat# 13–0700, Invitrogen, Carlsbad, CA). For controls, non-immune mouse IgM and rabbit IgG were used as primary antibodies, which yielded no detectable signals (**Fig. S2)**. Slides were washed three times with PBS and incubated with Alexa 555-conjugated goat anti-mouse IgG or Alexa 555-conjugated goat anti-rabbit IgG (1∶800, Jackson Immunoresearch, West Grove, PA) for 2 h at room temperature. Sections were then washed with PBS, and some sections were incubated with diamidino-phenylindole (DAPI) for 10 min, rewashed with PBS, mounted with Aqua Poly/Mount medium (Polysciences Inc, Warrington, PA), and tile imaged with an Axiovert200 Fluorescence Live Cell Imaging Workstation (Zeiss).

### Quantification of Immunofluorescence

Fluorescence intensities of spinal cord areas immunolabeled for CS56 (*n* = 3), C4S (*n* = 4), GFAP (*n* = 3), TH (*n* = 4), 5-HT (*n* = 5), and Iba1 (*n* = 3) were quantified using four sagittal sections spaced 400 µm apart, per condition. Photographic documentation was performed with the Axiovert200 Fluorescence Live Cell Imaging Workstation (Zeiss), AxioVision software (Zeiss) and Image J software. Both, immunostaining and imaging were performed under identical conditions by an experimenter blinded to the type of treatment. Staining intensity thresholds for each antibody were determined after all images were acquired to optimize the signal-to-noise ratio for each antibody. The threshold selected was 55 (within the full range of intensities extending from 0 to 255) for CS56-immunoreactivity (IR), 55 for C4S-IR, 55 for GFAP-IR, 40 for TH-IR, 40 for 5-HT-IR and 55 for Iba1-IR. The mean fluorescence intensity of the area of IR at 0.4 mm equidistant or 1 mm caudal from the center of the injury site with intensities higher than the thresholds stated above, were measured and normalized to total tissue areas.

### Statistical Analysis

All numerical data are presented as group mean values with standard error of the mean (SEM). The statistical significance of BMS score and mean immunoreactivity intensity for each group was estimated by analysis of variance (one-way ANOVA), followed by Tukey’s *post-hoc* test. The p values <0.05 were considered statistically significant.

## Supporting Information

Figure S1 **Chondroitin 4-O-sulfate (C4S) immunoreactivity is reduced by acute injection of ARSB after moderate compression injury.** Immediately after spinal cord injury, one µl of ARSB (10 U/ml) was injected at the injury site (arrows) and 0.5 mm rostral and caudal to this site (arrowheads). After 5 days, the mice were perfused, and sagittal spinal cord sections were analyzed by immunofluorescence using an antibody specific for C4S. Except for the meninges, no immunoreactivity is detectable in sham-operated control animals (A). C4S immunoreactivity is higher at the injury site in the buffer-injected control mice (B) versus ARSB-injected mice (C).
(TIF)

Figure S2 **Immunoreactivity of IgM and IgG isotype controls from non-immune mice (A) and rabbits (B), respectively.** Fluorescence staining in sagittal sections at the injury site (arrow) and at the caudal and rostral injection sites (arrowheads) 9 weeks after control injected group, being less pronounced than the immunoreactivity seen with the specific immune antibodies as shown in **Fig. S1** and **Figs. 2**, **3**, **4**, and **6** **.**
(TIF)
