# Supplementary figures and images for: Arylsulfatase B Improves Locomotor Function after Mouse Spinal Cord Injury
Source: PLoS One. 2013 Mar 8;8(3):e57415. doi: 10.1371/journal.pone.0057415 (PMC3592852; doi:10.1371/journal.pone.0057415)

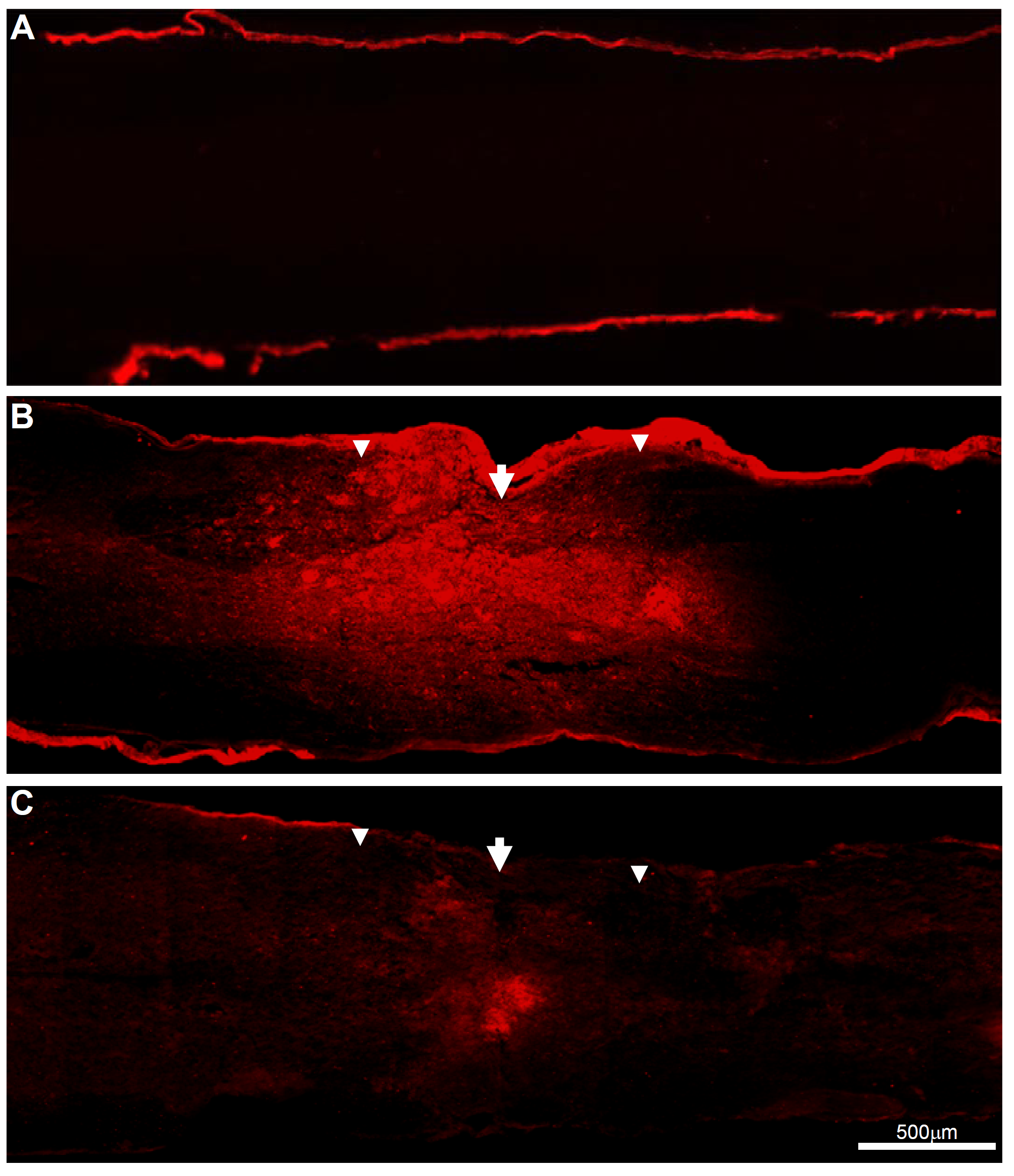

Supplement: Figure S1 — Chondroitin 4-O-sulfate (C4S) immunoreactivity is reduced by acute injection of ARSB after moderate compression injury. Immediately after spinal cord injury, one µl of ARSB (10 U/ml) was injected at the injury site (arrows) and 0.5 mm rostral and caudal to this site (arrowheads). After 5 days, the mice were perfused, and sagittal spinal cord sections were analyzed by immunofluorescence using an antibody specific for C4S. Except for the meninges, no immunoreactivity is detectable in sham-operated control animals (A). C4S immunoreactivity is higher at the injury site in the buffer-injected control mice (B) versus ARSB-injected mice (C). (TIF) [file pone.0057415.s001.tif]

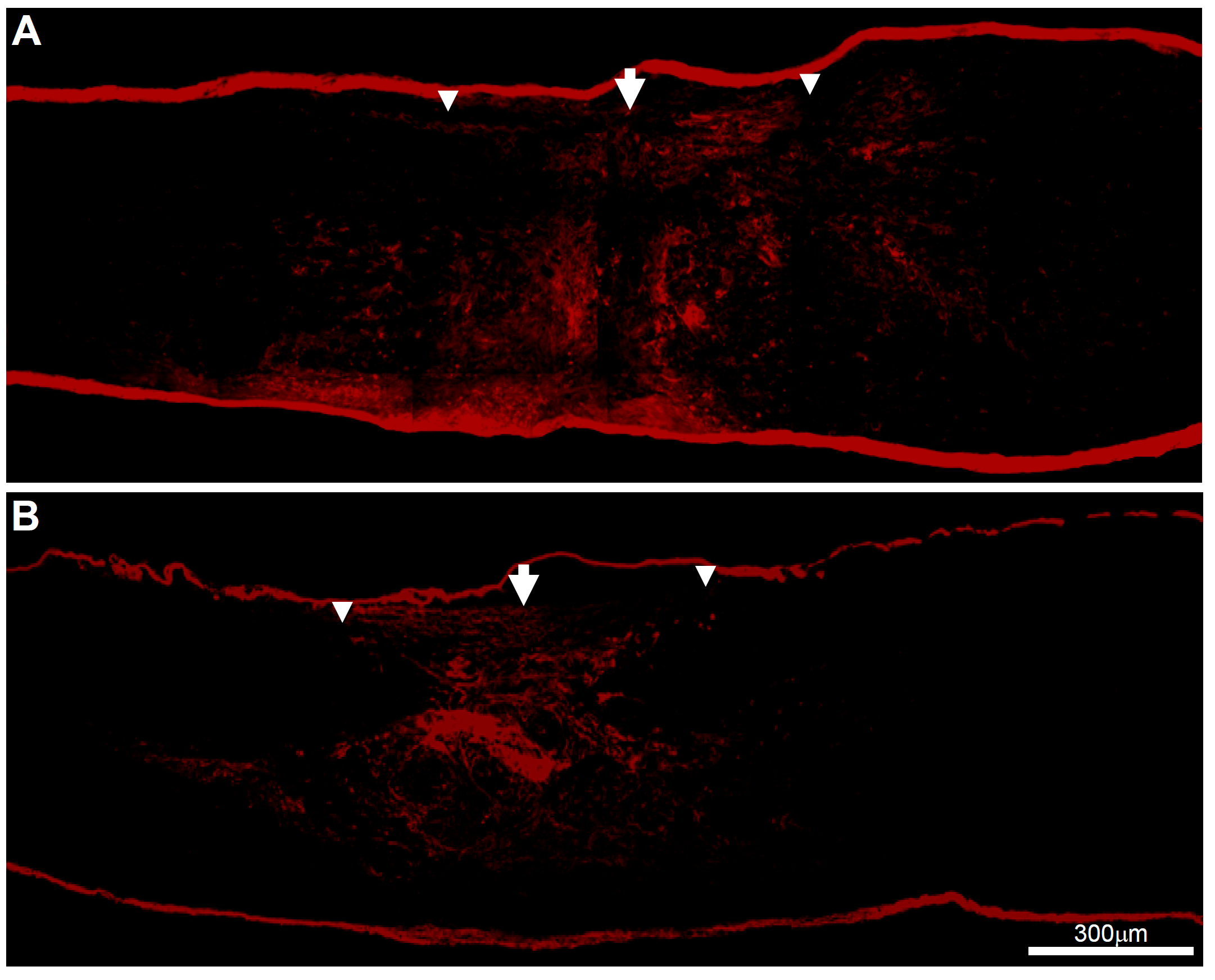

Supplement: Figure S2 — Immunoreactivity of IgM and IgG isotype controls from non-immune mice (A) and rabbits (B), respectively. Fluorescence staining in sagittal sections at the injury site (arrow) and at the caudal and rostral injection sites (arrowheads) 9 weeks after control injected group, being less pronounced than the immunoreactivity seen with the specific immune antibodies as shown in Fig. S1 and Figs. 2 , 3 , 4 , and 6 . (TIF) [file pone.0057415.s002.tif]
